# Quality of Life Following the Surgical Management of Gastric Cancer Using Patient-Reported Outcomes: A Systematic Review

**DOI:** 10.3390/curroncol31020065

**Published:** 2024-02-04

**Authors:** Patrick Cullen Vallance, Lloyd Mack, Antoine Bouchard-Fortier, Evan Jost

**Affiliations:** 1 Department of Surgery, University of Calgary, Calgary, AB T2N 1N4, Canadaantoine.bouchard-fortier@albertahealthservices.ca (A.B.-F.);; 2 Department of Oncology, University of Calgary, Calgary, AB T2N 1N4, Canada

**Keywords:** quality of life, QoL, patient-reported outcome measure, PRO, gastric cancer, stomach neoplasm, gastrectomy, gastric resection

## Abstract

**Introduction:** Surgical management of gastric adenocarcinoma can have a drastic impact on a patient’s quality of life (QoL). There is high variability among surgeons’ preferences for the type of resection and reconstructive method. Peri-operative and cancer-specific outcomes remain equivalent between the different approaches. Therefore, postoperative quality of life can be viewed as a deciding factor for the surgical approach. The goal of this study was to interrogate patient QoL using patient-reported outcomes (PROs) following gastrectomy for gastric cancer. **Methods:** This systematic review was registered at Prospero and followed PRISMA guidelines. Medline, Embase, and Scopus were used to perform a literature search on 18 January 2020. A set of selection criteria and the data extraction sheet were predefined. Covidence (Melbourne, Australia) software was used; two reviewers (P.C.V. and E.J.) independently reviewed the articles, and a third resolved conflicts (A.B.F.). **Results:** The search yielded 1446 studies; 308 articles underwent full-text review. Ultimately, 28 studies were included for qualitative analysis, including 4630 patients. Significant heterogeneity existed between the studies. Geography was predominately East Asian (22/28 articles). While all aspects of quality of life were found to be affected by a gastrectomy, most functional or symptom-specific measures reached baseline by 6–12 months. The most significant ongoing symptoms were reflux, diarrhoea, and nausea/vomiting. **Discussion:** Generally, patients who undergo a gastrectomy return to baseline QoL by one year, regardless of the type of surgery or reconstruction. A subtotal distal gastrectomy is preferred when proper oncologic margins can be obtained. Additionally, no one form of reconstruction following gastrectomy is statistically preferred over another. However, for subtotal distal gastrectomy, there was a trend toward Roux-en-Y reconstruction as superior to abating reflux.

## 1. Introduction

Surgical treatment for gastric adenocarcinoma remains the only modality offering a definitive cure [1]. The tumour location, type, and infiltrative pattern dictate what type of resection is offered, either proximal (PG), distal (DG), or total gastrectomy (TG) with concomitant D2 lymphadenectomy [2]. With accompanying peri-operative chemoradiation, a gastric resection can have a drastic effect on a patient’s quality of life. Over the last 30 years, many instruments have been validated to help measure the impact of gastric resection on quality of life using patient-reported outcomes (PROs). The most widely used, demonstrating cross-cultural applicability, is the European Organization for Research and Treatment of Cancer (EORTC) QLQ-C30, which was designed for cancer-specific patient quality-of-life outcome measures [3,4]. The EORTC QLQ-STO22 questionnaire supplements the QLQ-C30 by measuring gastric cancer-specific outcomes, such as dysphagia, eating restrictions, pain, and reflux [5,6].

The ultimate goal of surgery Is a radical, margin-free gastric resection and an optimal functional outcome. While oncologic considerations dictate the extent of resection, the type of reconstruction remains far more varied. Billroth I (B-I), Billroth II (B-II), and Roux-en-Y (RY) operations encompass the three broad categories of reconstruction after subtotal (SG) and total gastrectomy. However, there are many differences in how each can be performed. This creates uncertainty regarding which reconstructive method is best. Typically, surgeon preference dictates the type of reconstruction.

In the North American context, little is known about how differences in reconstruction are related to quality of life. The aim of this systematic review was to investigate patient quality of life using patient-reported outcomes following gastrectomy for gastric cancer, with a specific focus on reconstructive methods.

## 2. Methods

### 2.1. Protocol and Registration

The protocol for this review was registered in PROSPERO (https://www.crd.york.ac.uk/PROSPERO, accessed on 10 July 2020) with the number CRD42020177828.

### 2.2. Search Strategy

A systematic literature search of the MEDLINE, EMBASE, and SCOPUS databases was undertaken from inception to 18 January 2020. Additional studies from prior systematic reviews were manually imported [4,7]. Using MESH terms and search operators, we included the following three general concepts: gastric cancer, gastrectomy, and quality of life or patient-reported outcomes. The authors undertook a consultation with a librarian at the University of Calgary to ensure the accuracy and completeness of the search strategy. The exact search strategy used for MEDLINE is outlined in Appendix A.

### 2.3. Study Selection and Extraction

Relevant studies were imported into the COVIDENCE online software designed for systematic reviews (www.covidence.org, 18 January 2020). Two independent authors (P.C.V. and E.J.) independently assessed titles and abstracts for inclusion and full-text articles for eligibility. Any conflicts were resolved with an independent third reviewer (A.B.F.).

**Figure 1:**
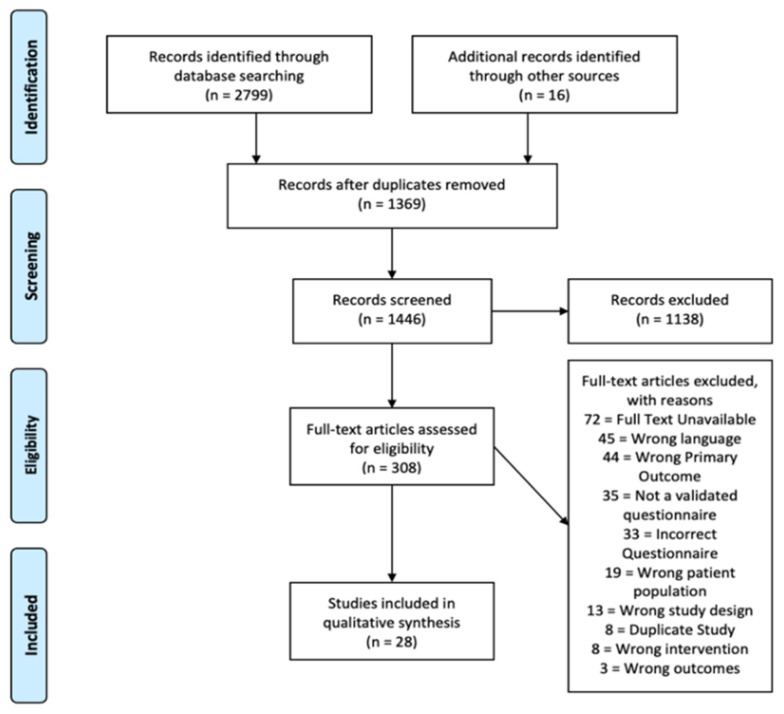
Summary and results of search strategy.

See Figure 1 for the PRISMA table.

Selection/eligibility criteria are outlined below.

(1) Articles published in English.
(2) Only full-text articles were included: No abstracts, letters to the editor, or case reports.
(3) Questionnaire: The article must investigate the quality of life through a patient-reported outcome questionnaire that has been validated by the European Organization of Research and Treatment of Cancer, Generic (QLQ-C30 or QLQ-C36) and/or Site-specific (STO-22). Validation was determined through the identification of previous literature measuring the clinical and psychometric reliability of the PRO instrument across multiple languages.
(4) Pathology: Only gastric carcinomas were included; neuroendocrine, GIST, lymphoma, and benign tumours were excluded.
(5) Resection: Subtotal and total gastrectomies were included. The subtotal included proximal and distal gastrectomy. Wedge and local resections were excluded.
(6) Surgical technique: Open and minimally invasive approaches were included.
(7) Reconstruction: All methods were included.
(8) Population: Age higher than 18.

### 2.4. Data Items and Extraction

Data were extracted using a standardized, pre-defined collection form in Microsoft Excel (2019). Study details, publication date, country, study design, type of gastrectomy, type of reconstruction, number of patients included, length of follow-up, response rate, and conclusion were included. Studies were separated based on their research purpose, either to detect differences in QoL between types of resection, methods of reconstruction, or open vs. laparoscopic approaches. The authors were not contacted for raw data as a meta-analysis was not performed. A summation of study results and frequencies was performed, and no advanced statistical analysis was performed.

### 2.5. Questionnaire

The choice of the European Organization for Research and Treatment of Cancer (EORTC) Generic (QLQ-C30 or QLQ-C36) and Site-specific (STO-22) questionnaires in this systematic review was driven by the need for a widely accepted and extensively used quality-of-life instrument. The EORTC tools have established themselves as reliable measures of health-related quality of life in cancer patients, facilitating consistent comparisons across various studies and worldwide populations. Our decision to opt for the EORTC questionnaire was rooted in the desire to ensure the reliability and comparability of results across the studies that would be examined. Alternative tools like the Postgastrectomy Syndrome Assessment Scale (PGSAS-45), the Gastrointestinal Quality of Life Index (GIQLI), or the Functional Assessment of Cancer Therapy (FACT-Ga) scale were considered. While these do have specific strengths, the general symptomatology captured by each of them is similar. We were cautious about using a wide range of questionnaires interchangeably due to concerns about the lack of reliable comparability. The EORTC questionnaires, with their established psychometric properties and widespread adoption, provide a solid foundation for meaningful cross-study comparisons and contribute to the overall robustness of the conclusions drawn. The use of the EORTC would also provide a basis for data extraction for possible future meta-analyses.

## 3. Results

### 3.1. Study Selection

The results of the search strategy are outlined in Figure 1; of the 1446 studies identified, 28 were selected for analysis.

### 3.2. Study Methodology

Length and method of follow-up were variable, ranging from 1 to 81 months. A prospective study design predominated with 16 studies out of the total 28 [8,9,10,11,12,13,14,15,16,17,18,19,20]. Thirteen studies were able to capture long-term data (greater than 2 years of follow-up) [8,12,17,21,22,23,24,25,26,27,28,29,30].

### 3.3. Study Aims and Outcomes

The aim of each study could be broadly separated into the patient’s quality of life as it relates to three categories of surgical approach: resection (Total vs. Subtotal gastrectomy), reconstruction (RY vs. B-I vs. B-II, Jejunal Interposition and Pouches, and Pylorus Preserving), and open vs. laparoscopic.

### 3.4. Questionnaire

All studies except one used the more general questionnaire, EORTC QLQ-C30, developed to assess the quality of life of cancer patients [31]. Twenty-two studies used the site-specific questionnaire, EORTC QLQ-STO22. In conjunction with the above, other administered questionnaires include Kuchler, QLQ-OES18, GSRS, and Daugs-20 [16,23,25,32].

### 3.5. Resection

#### 3.5.1. Late Outcomes (≥2 Years of Follow-Up)

Four of six studies commented that the global quality of life following a total gastrectomy was equivalent to a subtotal gastrectomy [8,21,22,23,31,33]. Two of these four studies analysed distal gastrectomy (DG), and the other two did not distinguish the type of subtotal gastrectomy [22,23]. No studies showed a statistically significant difference in global QoL between the SG and TG groups over the past two years of follow-up. However, all studies comment on statistically significant higher symptom scores in the TG group. Total gastrectomy scored higher (i.e., worse) in eating restrictions, dysphagia, nausea/vomiting, reflux, and diarrhoea. For example, Goh et al. showed symptom scores were on average 37.1% worse for TG [33]. The symptoms that were statistically significant were variable across the studies; however, eating restriction was the most reported negative symptom associated with TG. Given the higher symptom scores, two studies concluded that a subtotal gastrectomy has a superior QoL [21,31].

#### 3.5.2. Early Outcomes (<2 Years of Follow-Up)

Five of six studies suggest that in the early post-operative period, subtotal gastrectomy is superior to a total gastrectomy in terms of quality of life [8,9,10,11,12,21]. All studies showed that symptoms improved nearly to their baseline between 6 and 12 months. One study compared types of SG (proximal and distal), which showed no difference between TG and DG; however, these were both superior to PG [11]. Two studies did not state which type of SG was performed [10,21], and three compared DG to TG [11,31].

Karanicolas et al. noted that all gastrectomy patients immediately post-operatively suffer a 50–70% impairment to their global QoL, physical and role functioning [11]. Most patients had significant improvement in their symptoms over time. However, 20–35% continued to have substantially worse functioning, persisting to the last assessed follow-up at 18 months post-operatively [11].

### 3.6. Reconstruction Method

Among those reviewed, there were 13 studies that aimed to study how the post-gastrectomy reconstructive method influenced quality of life [13,14,15,16,17,18,21,24,25,26,27,34,35]. Rausei, in addition to analysing TG vs. SG, also examined the effect of RY vs. BII, as such was included [21].

#### 3.6.1. Roux-en-Y vs. Billroth I vs. Billroth II

Six studies analysed the reconstructive methods of RY, B-I, and B-II [16,18,21,25,34,35] (Table 1). In terms of global QoL, only one of six studies found a significant difference between any reconstruction type. Yang et al. compared BI vs. RY and found that at one year, RY was superior in terms of global health status (RY 88.8 vs. B-I 85.4) and had lower pain scores than B-I [18].

#### 3.6.2. Pouch

Four studies analysed how various pouch reconstructions would influence QoL [13,14,17,24]. Huang noted that pouch reconstruction with either R-Y or BII led to a marginal symptom improvement in nausea, emesis, and appetite loss, though this did not greatly contribute to an individual’s overall QoL [24]. Hoksh et al. showed a trend toward improved QoL with a larger pouch [13]. Tanaka et al. did note that an aboral pouch following RY appeared to alleviate the symptom of diarrhoea which was significant at 5 years post-op [17].

#### 3.6.3. Jejunal Interposition (JIP)

Two studies looked at jejunal interposition [13,27]. Namikawa et al. state that JIP reconstruction had improved short-term QoL outcomes, showing improved global health status (JPI 80.6 vs. RY 54.4) and physical functioning, as well as lower dyspnoea, insomnia, and diarrhoea [27]. However, this impact decreased over time, with only the symptom of fatigue being significantly in favour of JIP at 5 years [27]. While Hoksch et al. analysed JIP, the comparison groups were various sizes of interposition pouches [13]. As such, it was difficult to comment on how this technique compares to other reconstructions. 

### 3.7. Laparoscopic Approaches

Five studies compared a laparoscopic-assisted vs. open approach [11,19,28,29,32]. Generally, the majority found that the laparoscopic-assisted one is superior to an open approach [11,19,32]. Of note, the follow-up for these studies was a maximum of one year. In one study that examined patients at two years post-surgery, the authors commented that a laparoscopic-assisted distal gastrectomy did not have an advantage over an open approach [28]. Two other studies compared a totally laparoscopic approach against a laparoscopic-assisted approach [20,30]. Both studies had a short follow-up after surgery, three and six months [20,30]. One of these concluded that a totally laparoscopic approach had improved pain and dysphagia [30]. Two studies also focused on a laparoscopic pylorus-preserving approach as it relates to QoL [26,35].

### 3.8. Additional Analyses

Bae et al. (2006) found that chemotherapy and radiotherapy did not affect QoL scores. Additionally, they investigated preoperative predictors of QoL and found that any comorbidity, lower education, younger age, and female patients had a propensity toward lower QoL [22].

Diaz de Liano et al. (2003) found that D1 vs. D2 and major post-operative complications had no bearing on QoL [23].

## 4. Discussion

A proper oncologic resection should be the most important priority in surgical decision-making. The studies in this systematic review, which directly analysed subtotal vs. total gastrectomy, comment on a “less is more” approach whereby subtotal gastrectomy created an overall QoL benefit. However, this was not statistically significant over time. There were six studies that commented on late outcomes (follow-up more than 2 years), and all these studies state that there are select symptom scores that were statistically worse for a total gastrectomy. Only two of these went on to conclude that a subtotal gastrectomy is superior to a total gastrectomy [11,16]. Ultimately, there is little difference between reconstructive methods. Roux-en-Y reconstruction does appear to provide a more favourable outcome in terms of reflux than either Billroth-I or Billroth-II (Table 1).

Most of the studies included in this review were performed on Asian populations. This is likely due to the higher incidence of gastric cancer in these areas, increasing the opportunity for high-volume studies. We expected to find a negative impact on quality of life related to any extent of gastrectomy. Overall, the studies analysed corroborated this, with all studies reporting a decrease in one or more domains (physical, role, social, etc.) of functioning and an increase in negatively associated symptoms (dysphagia, reflux, etc.) from their pre-operative state to the closest follow-up time point post-operatively (on average of all studies, 3 months). However, these measures tended to return to their pre-operative baseline at six months to one year. While the authors attempted to quantify the exact magnitude of the decrease in quality of life, this was confounded by the heterogeneity of study design, resection and reconstruction type, and length of follow-up. Interestingly, some of the studies found that global QoL increased in a linear fashion post-operatively. The authors of these studies concluded that this may be related to a negative pre-operative emotional state and the relief patients felt at having had their cancer resected [16]. There are many additional factors that contribute to a patient’s symptoms and functioning. Very few of the studies commented on the effect of chemotherapy and radiotherapy on QoL. These are important potential confounders in the understanding of QoL, which few studies comment on. We speculate that the increase in QoL over time post-operatively may be related to the conclusion of peri-operative adjuvant treatments. Kim et al. and Zieran et al. mention that fatigue related to chemotherapy could be the most significant contributor to a patient’s symptoms and can have a profound effect on a patient’s QoL and role functioning [10,36].

The majority of the studies focused on resection and analysed distal gastrectomy versus total gastrectomy. However, there were several studies which did not mention what type of gastrectomy was performed. These studies noted a subtotal gastrectomy was performed, though they did not specify if it was proximal or distal. This unfortunately detracts from study quality, as a QoL difference between proximal and distal gastrectomy is supported by Karanicolas et al. [9]. In the interval period from when this review was conducted and written, several papers were published comparing these reconstructive approaches. These suggest, as we have found, that there is little to no difference in overall QoL between RY, B-I, and B-II [37,38]. However, there is a slight difference in bile reflux gastritis and reflux esophagitis favouring RY reconstruction [37,38]. This may be the reason that RY has a slight statistical advantage in terms of symptoms, as it can prevent bile acid reflux by making the bile join the alimentary tract distal to the gastric remnant. These studies were not retrospectively included in our systematic review in order to maintain the academic integrity of the search strategy. However, these papers are available within the references for further review.

Similar to the “less is more” discussion of SG vs. TG, a minimally invasive resection is favoured over an open surgery in the first six months post-operatively. This near-term benefit tends to wane over time with the two approaches becoming equivalent from six months to one year. Misawa et al. undertook a multi-institutional nonrandomised study, which found that a laparoscopic distal gastrectomy had improved symptom scores that reached non-significance by six months [19]. However, this group developed improved role, emotional, and cognitive functioning that became significant only after six months [19]. Interestingly, one study determined an open distal gastrectomy was superior to a laparoscopic approach [29]. The authors explicitly comment that this was unexpected, and this finding may be associated with the patient’s inappropriately high expectation of a laparoscopic surgery [29].

The scope of this systematic review did not permit the examination of post-operative complications or surgical outcomes as they relate to the various methods of gastric resection and reconstruction. Many other studies have suggested that length of stay, anastomotic leakage, delayed gastric emptying, and post-operative morbidity/mortality are relatively equivalent between resection and reconstructive methods [37,38,39,40]. However, this has a low level of evidence as there are limited high-quality randomised control trials allowing for a strong conclusion.

In this review, the EORTC was selected as the desired questionnaire as it is the most ubiquitous and widely validated [4]. However, it is important to note that other questionnaires do exist, e.g., PGSAS-45, GSRS, and GIQLI [4]. While these questionnaires measure PROs, they do so with different questions and grading systems. Conceptually, this might cause the same patient to answer differently depending on the questionnaire provided. This impairs the validity of comparing PROs across different studies. Therefore, while outlining our methodology, we elected to limit the selected studies to the EORTC questionnaires. While focusing on the EORTC questionnaire allowed us to make more robust comparisons, it also introduced a limitation in that there may have been valuable studies examining post-gastrectomy QoL that were ultimately excluded because they did not utilize the EORTC questionnaire. In the review of the literature, we identified the PGSAS-45 questionnaire as a robust alternative to the EORTC group. This questionnaire was developed by the Japanese Postgastrectomy Syndrome Working Party, which was charged with developing a specific QoL questionnaire to examine this patient population [41]. This survey brought together the Short-Form Health Survey (SF-8) and the Gastrointestinal Symptom Rating Scale (GSRS), in addition to specific questions about food intake, gastrointestinal upset, and dumping syndrome [41]. This survey has been widely used in the Japanese population. An area for future study would be to validate and apply this to North American or European populations.

Initially, as part of our methodology, we had planned to perform a meta-analysis. This was another factor in our reasoning for selecting the EORTC questionnaire—to have concordance with the reported results. However, the studies that were analysed in this systematic review had significant heterogeneity. The discrepancy in follow-up and reported results did not allow us to identify, tabulate, and pool the various outcome measures of the EORTC questionnaires. We also felt there were not enough studies within each subcategory to constitute a true sample of a larger population, and therefore could not be considered a random sample. A biostatistician was consulted, who confirmed that amalgamating our available data into a meta-analysis would result in poor confidence and low-quality conclusions. This is an additional limitation of our review.

In conclusion, while necessary, a resection for gastric cancer may negatively impact a patient’s quality of life. Despite the initial negative impact, most studies suggest that overall QoL returns to baseline at six months to one year post-operatively. A subtotal gastrectomy and laparoscopic approach should be chosen if oncologic parameters allow for such a resection. There is no preferred method of reconstruction, although if there is a choice, RY reconstruction may provide benefits with respect to reflux-related symptoms. Ultimately, surgeons should choose the reconstructive method that they are most experienced with. Additionally, this systematic review highlights the heterogeneity within the field of gastric resection and reconstruction as it relates to PROs. Future quality-of-life studies should aim to standardize follow-up intervals and reported results and utilize a single validated questionnaire. This would help clarify many of the limitations of this research, allowing for more robust conclusions.

## Figures and Tables

**Table 1:** Summary of studies comparing B-I, B-II, and RY reconstructions.

First Author	Year of Publication	Ref.	Country	Study Type	Number of Patients	Length of Follow-Up	Comparison Group	Results		Author’s Conclusion
								Global QoL	Significant Differences	
Huang	2007	[24]	Taiwan	Retrospective, cross-sectional	51	Avg. 17 mos (range 6–24 mos)	Subtotal Gastrectomy and BII vs. Total Gastrectomy and RY ± pouch	Does not depend on the stage of cancer (early 66.7 vs. late 66.7) or resection (SG 67 vs. TG 67).	SG>TG: role function, N/V, and appetite loss. No obvious QoL advantages to either HLR pouch or braun jejunojenuostomy.	“Gastric adenocarcinoma survivors may enjoy a similar life quality, regardless of their original disease stages. Functional preservation may have marginal advantages to improve the patients’ quality of life by reducing symptomatic nausea, vomiting, and appetite loss postoperatively.”
Takiguchi	2012	[25]	Japan	Prospective, RCT	327	Avg. 21 mos (range 3–34 mos)	All patients had subtotal gastrectomy; BI vs. RY	Global health status similar in both groups (B-I 73.5 ± 21.3, R-Y 73.2 ± 20.2, *p* = 0.87).	RY is better on the dyspnoea scale (B-I 13.6 ± 17.9, R-Y 8.6 ± 16.3, *p* = 0.02).	“The B-I and R-Y techniques were generally equivalent in terms of postoperative QOL and dysfunction. Both procedures seem acceptable as standard reconstructions after distal gastrectomy with regard to postoperative QOL and dysfunction.”
Rausei	2013	[21]	Italy	Retrospective, cohort	103	Avg. 81 ± 80.7 mos (range 2–300 mos)	Total vs. Subtotal gastrectomy; SG w/BII vs. SG w/RY, D1 vs. D2	RY group had a number and relative percentage of patients who had a higher score for health status and QoL (score range 5–7).	RY better for symptoms related to dumping syndrome: need for resting after eating, discomfort during meals, and symptoms related to abdominal distention.	“QoL after gastric surgery for cancer is affected by tumour- and treatment-related factors. In order to improve patients’ QoL, subtotal resection with Roux-en-Y reconstruction should be preferred whenever oncologically acceptable.”
Smolskas	2015	[34]	Lithuania	Retrospective, cohort	266	6–12 mos vs. >12 mos	All patients had subtotal gastrectomy; BI vs. Balfour vs. RY	No difference. B-I (62 +/− 20), B-II (56 +/− 21), and RY 61 +/− 24).	No significant difference between any reconstruction type or post-operative duration.	“The best QoL scores were obtained from the patients who underwent the Billroth I surgery. The Roux-en-Y method was better than the Balfour method 6–12 months after surgery. However, the Balfour method was better than the Roux-en-Y after one year. Further prospective randomised controlled trials are needed.”
Yang	2017	[18]	China	Prospective, RCT	136	Baseline, 3, 6, 9, and 12 mos	All patients had subtotal gastrectomy; BI vs. RY	RY > B-I at 1 year (88.8 RY vs. 85.4 B-I).	RY lower reflux symptoms at 6mos and 9 mos; non sig. at 1 year. RY lower pain score at 1 year.	“Both B-I and R-Y anastomosis are safe and feasible which could be applied in clinical practice. The stronger anti-reflux capability of R-Y anastomosis contributes to a higher QoL by reducing the reflux-related gastritis and pain symptoms, and promoting better global health.”
So	2018	[16]	Singapore	Prospective, RCT	162	6 and 12 mos	All patients had a subtotal gastrectomy; BII vs. RY	No difference between B-II and RY at 1 year (71.6 vs. 73.8).	No differences between groups.	“BII is associated with a higher incidence of heartburn symptom and higher median endoscopic grade for gastritis, BII and RY are similar in terms of overall GI symptom score and nutritional status at 1 year after distal gastrectomy.”
